# Supplementary material for: Treatment, Prognostic Markers, and Survival in Thymic Neuroendocrine Tumors, with Special Reference to Temozolomide-Based Chemotherapy
Source: Cancers (Basel). 2024 Jul 10;16(14):2502. doi: 10.3390/cancers16142502 (PMC11275075; doi:10.3390/cancers16142502)
Supplement: Supplementary file 1 [file cancers-16-02502-s001.zip › cancers-3079232-supplementary.pdf]

Table S1 Individual patient data about clinicopathological characteristics.

ID	Age (years)	Gender	Histological diagnosis	Mitotic index (10HPF)	Tumor size (cm)	TNM stage	Masaoka stage	Hematogenous metastases		MEN1 syndrome	MGMT
								At diagnosis	During disease		
1	56	male	AC	9	5.1	I	IIa	/	/	No	-
2	33	male	AC	2	6.0	I	I	/	Lung, Cervical lymph node, Mediastinum, Supraclavicular lymph node	No	-
3	47	male	AC	8	4.0	I	IIa	/	/	No	-
4	35	female	AC	2	6.2	IVb	IVb	Brain	Brain	No	-
5	56	male	TC	2	5.7	IVb	IVb	Lung, Bone, Mediastinum	Lung, Bone, Mediastinum, Liver, Spleen	No	-
6	55	male	AC	2	10.0	IVa	IVb	Cervical lymph node	Cervical lymph node, Supraclavicular lymph node, Pancrease	No	+
7	44	male	AC	1	12.0	IIIb	III	/	/	No	+
8	50	female	HG-NET	20	5.0	IIIa	III	/	Supraclavicular lymph node	No	+
9	30	female	HG-NET	22	5.0	IVb	IVb	Bone, Mediastinum, Supracla-vicular lymph node, Liver	Bone, Mediastinum, Liver, Supraclavicular lymph node	No	+
10	51	female	AC	2	7.0	I	I	/	/	No	+
11	46	male	AC	2	4.0	I	IIb	/	Cervical lymph node, Mediastinum	No	+
12	60	male	AC	6	2.0	I	IIb	Cervical lymph node	Cervical lymph node	No	+
13	57	male	AC	1	15.0	IVb	IVb	Bone	Bone, Mediastinum	No	+
14	44	male	AC	2	6.0	I	IIb	/	Lung, Bone, Cervical lymph node	No	+
15	45	male	AC	2	7.0	IIIa	III	/	Lung, Bone, Mediastinum, Supraclavicular lymph node	No	+

Table S1. (to be continued)

ID	Age (years)	Gender	Histological diagnosis	Mitotic index (10HPF)	Tumor size (cm)	TNM stage	Masaoka stage	Hematogenous metastases		MEN1 syndrome	MGMT
								At diagnosis	During disease		
16	48	male	AC	10	8.1	IVb	IVb	Bone, Liver	Lung, Bone, Liver	No	+
17	52	male	AC	3	6.0	I	IIb	/	Mediastinum	No	+
18	45	male	AC	6	8.0	IVb	IVb	Lung, Cervical lymph node	Lung, Cervical lymph node, Supraclavicular lymph node	Yes	+
19	49	male	AC	4	5.0	IVb	IVb	Mediastinum, Supraclavicular lymph node	Bone, Mediastinum, Supraclavicular lymph node	Yes	+
20	50	male	AC	2	10.0	I	I	/	/	Yes	+
21	57	male	HG-NET	15	11.0	IIIb	III	/	Cervical lymph node, Mediastinum	No	+
22	37	male	TC	1	6.0	IIIb	IVb	Bone	Bone	No	+
23	22	female	AC	2	4.3	IVb	IVb	Lung, Bone, Mediastinum	Lung, Bone, Mediastinum	No	+
24	58	male	AC	9	6.5	I	I	/	Bone, Mediastinum	No	+
25	47	male	AC	5	7.0	I	I	/	/	No	+
26	29	female	HG-NET	20	6.0	I	I	/	Mediastinum	Yes	+
27	66	male	AC	8	4.5	IIIa	III	/	/	No	+
28	43	male	AC	10	10.2	IVa	IVb	/	Mediastinum	No	+
29	63	female	AC	1	5.0	I	I	/	Cervical lymph node, Thyroid	No	+
30	43	male	TC	1	6.1	IVb	IVb	Bone, Cervical lymph node, Supraclavicular lymph node	Bone, Cervical lymph node, Supraclavicular lymph node	Yes	+
31	51	female	AC	8	11.0	I	IIb	/	Bone, Liver	Yes	+
32	38	male	AC	2	2.0	IVb	III	Lung	Lung, Bone, Mediastinum, Pericardium	No	+

TC: typical carcinoid; AC: atypical carcinoid; HG-NET: Carcinoids/NETs with elevated mitotic counts and/or Ki67 proliferation index； MEN1: multiple endocrine neoplasia type 1; MGMT: oxygen 6-methylguanine-DNA methyltransferase; +: positive； -: negative.

Table S2. Individual patients' data about treatment.

ID	OP	Postoperative treatment	Medical treatment	Treatment cycles	RT dose	Recurrence	DFS (m)	PFS (m)	OS (m)	Survival status	Treatment regimens after recurrence/progressive
1	+	None	/	/	/	-	49.2	49.2	48.6	die	/
2	+	C+RT	Etoposide + Cisplatin	4	4	+	50.8	50.8	86.3	alive	Etoposide + Cisplatin
3	+	C	Etoposide + Cisplatin	4	/	-	28.3	28.3	49.1	alive	/
4	-	/	Capecitabine + Temozolomide	12	13	/	/	11.6	32.1	alive	Oxaliplatin + Surufatinib[a]
5	-	/	Etoposide + Cisplatin	2	/	/	/	2.3	63.4	alive	Apatinib
6	-	/	Etoposide + Cisplatin	6	35	/	/	61.0	123.7	die	Capecitabine +Temozolomide
7	-	/	Capecitabine + Temozolomide	5	/	/	/	5.2	12.3	die	/
8	+	C	Etoposide + Cisplatin	4	/	+	74.4	74.4	83.7	alive	Surufatinib
9	-	/	Etoposide + Cisplatin	3	/	/	/	3.1	11.2	die	Capecitabine + Temozolomide
10	+	None	/	/	/	+	/	54.8	74.4	alive	/
11	+	None	/	/	/	+	16.0	16.0	106.5	alive	Octreotide acetate microspheres
12	+	C	Etoposide + Cisplatin	4	/	-	68.5	68.5	69.7	alive	/
13	-	/	Etoposide + Cisplatin	5	/	/	/	4.9	55.8	die	Capecitabine + Temozolomide
14	+	C+RT	Gemcitabine + Cisplatin	2	20	-	71.8	71.8	98.3	die	Octreotide acetate microspheres
15	+	None	/	/	/	+	23.2	23.2	85.2	alive	Capecitabine + Temozolomide
16	-	/	Capecitabine + Temozolomide	11	/	/	/	12.5	42.7	die	Surufatinib + Tislelizumab
17	+	C	Etoposide + Carboplatin	4	/	+	17.4	17.4	48.2	alive	Capecitabine + Temozolomide
18	-	/	Octreotide acetate microspheres	4	/	/	/	4.6	37.2	die	Etoposide + Carboplatin
19	+	C	Etoposide + Carboplatin	4	/	+	11.3	11.3	133.0	alive	Octreotide acetate microspheres
20	+	None	/	/	/	+	48.2	48.2	102.0	alive	RT
21	+	None	/	/	/	+	3.4	3.4	36.2	alive	Irinotecan + Nedaplatin
22	-	/	Everolimus	/	/	/	/	5.2	41.6	die	Capecitabine + Temozolomide
23	-	/	Capecitabine + Temozolomide	4	/	/	/	4.1	34.3	alive	Capecitabine + Temozolomide

Table S2. (to be continued)

ID	OP	Postoperative treatment	Medical treatment	Treatment cycles	RT dose	Recurrence	DFS (m)	PFS (m)	OS (m)	Survival status	Treatment regimens after recurrence/progressive
24	+	None	/	/	/	+	32.5	32.5	91.3	die	Surufatinib
25	+	C	Etoposide + Cisplatin	4	/	-	58.0	58.0	58.0	alive	/
26	+	None	/	/	/	+	11.6	11.6	32.9	alive	Surufatinib
27	+	C	Capecitabine + Temozolomide	13	/	-	30.0	30.0	30.4	die	/
28	+	None	/	/	/	+	9.8	9.8	41.9	alive	Capecitabine +Temozolomide
29	+	None	/	/	/	+	67.3	67.3	94.6	alive	Octreotide acetate microspheres
30	-	/	Capecitabine + Temozolomide	14	/	/	/	25.1	35.4	alive	Surufatinib
31	+	RT	/	/	25	+	96.9	96.9	124.3	alive	Paclitaxel + Cisplatin
32	+	C+RT	Paclitaxel + Cisplatin	2	10	+	4.9	4.9	27.5	alive	Surufatinib

[a] Surufatinib's clinical research was mainly conducted in China, so it is only recommended in Chinese guidelines for the treatment of advanced Th-NETs.

[b] Tislelizumab is not currently recommended in clinical guidelines for the treatment of thymic neuroendocrine tumors (Th-NETs). However, given the rapid progression observed in the patient's tumor and genetic test results indicating potential responsiveness to immunotherapy, we initiated treatment with Tislelizumab following informed consent from the patient's family. This approach aligns with a personalized treatment strategy based on specific genetic insights.

Op: primary tumor surgery; DFS: disease-free survival; PFS: progression-free survival; OS: overall survival; C: Chemotherapy; RT: radiotherapy.
